# An eHealth-Delivered Total Diet Replacement Intervention for Type 2 Diabetes Remission: Single-Arm Pilot Study

**DOI:** 10.2196/93074

**Published:** 2026-10-06

**Authors:** Youssef Chninou, Johannes Edholm, Jenny Engvall, Sofia Wahlqvist, Claudia Schien, Anna Winkvist, Julia Otten

**Affiliations:** 1 Department of Public Health and Clinical Medicine, Umeå University, Daniel Naezens väg, Umeå, Västerbotten, 90737, Sweden, 46 0907850000; 2 Primary Care Dietitians, County Council of Vasterbotten, Umeå, Västerbotten, Sweden; 3 Clinical Research Centre, Umeå University, Umeå, Västerbotten, Sweden; 4 Department of Internal Medicine and Clinical Nutrition, University of Gothenburg, Gothenburg, Västra Götaland, Sweden

**Keywords:** type 2 diabetes mellitus, diabetes remission, weight loss, low-calorie diet, meal replacement, eHealth, mobile health, pilot studies

## Abstract

**Background:**

The Diabetes Remission Clinical Trial (DiRECT) demonstrated that remission of type 2 diabetes can be achieved through substantial weight loss using a total diet replacement (TDR) intervention combined with structured support. Whether this approach can be delivered exclusively via eHealth has not been well studied.

**Objective:**

This pilot study aimed to evaluate whether a TDR intervention delivered solely via eHealth could induce clinically meaningful weight loss and achieve type 2 diabetes remission.

**Methods:**

This was a single-arm, nonblinded, single-center pilot study conducted at the clinical research facilities of Umeå University Hospital. Individuals with type 2 diabetes diagnosed within the past 6 years were included. Participants followed a TDR (~850 kcal/d) for 12 weeks, and then gradually reintroduced food, with a total study duration of 6 months. Diabetes medications were discontinued at the start of the study. Daily body weight and fasting glucose were measured at home and transmitted to the study team via connected devices. All clinical support was delivered through video calls and chat. The 2 prespecified coprimary outcomes were a reduction in weight of 15 kg or more and remission of diabetes, defined as glycated hemoglobin below 6.5% without glucose-lowering medication, from baseline to 6 months. A 75 g oral glucose tolerance test and indirect calorimetry were performed at baseline and at 6 months to assess resting energy expenditure (REE) and respiratory quotient (RQ).

**Results:**

Ten participants (mean age 58.5, SD 7.4 y; mean BMI 35.3, SD 5.9 kg/m²; n=9 men and n=1 woman) were enrolled. After 6 months, 6 of 10 participants achieved the prespecified coprimary outcome of ≥15 kg weight loss, and all 10 participants achieved the prespecified coprimary outcome of diabetes remission. Mean weight loss was 16.9 (SD 5.4; *P*=.005) kg, and glycated hemoglobin decreased by 1.0% (SD 0.8%*; P*=.008). Daily home measurements showed a marked reduction in fasting glucose within the first week of the intervention. Median REE decreased by 19% (IQR 17%-22%), while median fasting RQ increased by 15% (IQR 5%-27%) and median post–glucose-load RQ increased by 12% (IQR 9%-18%) at 6 months.

**Conclusions:**

In this pilot study, an eHealth-delivered TDR intervention was associated with substantial weight loss and diabetes remission at 6 months without the use of glucose-lowering medication. These findings support further evaluation of fully eHealth-delivered dietary interventions in larger and longer-term randomized controlled trials.

## Introduction

Type 2 diabetes is a major global health challenge affecting approximately 422 million people worldwide, and its prevalence has increased rapidly over recent decades [1]. Obesity and physical inactivity are key drivers of the disease [2], which is associated with an increased risk of microvascular and macrovascular complications, reduced quality of life, and substantial health care costs [3].

In recent years, diabetes remission has become an accepted therapeutic goal. A joint consensus statement from the American Diabetes Association (ADA) and the European Association for the Study of Diabetes (EASD) defines remission as a glycated hemoglobin (HbA_1c_) below 6.5% without antidiabetic drugs during a follow-up of at least 3 months [4]. Evidence indicates that substantial weight loss is the principal driver of remission and can be achieved through intensive dietary interventions or bariatric surgery. The Diabetes Remission Clinical Trial (DiRECT) study, conducted in a predominantly White population, demonstrated that a total diet replacement (TDR) intervention of approximately 840 kcal/day for 3 to 5 months resulted in diabetes remission in46% (68/149) of participants at 1 year and 36% (53/149) at 2 years [5]. The Diabetes Intervention Accentuating Diet and Enhancing Metabolism-I (DIADEM-I) trial extended these findings by demonstrating similarly high remission rates in a younger Middle East and North African population with a shorter duration of type 2 diabetes [6].

In DiRECT, participants received intensive face-to-face follow-up in primary care, with visits every 2 weeks during the first 5 months and monthly visits thereafter [7]. Although effective, this approach is resource-intensive. eHealth interventions may provide a scalable and more accessible alternative. The World Health Organization (WHO) recognizes eHealth as a strategy to improve the quality, safety, and efficiency of health care delivery [8]. Meta-analyses have shown that eHealth interventions improve glycemic control in people with diabetes and support clinically meaningful weight loss [9,10]. Furthermore, the National Health Service (NHS) Type 2 diabetes Path to Remission Programme demonstrated that diabetes remission through calorie restriction can also be achieved in routine clinical practice, with much of the intervention delivered remotely through digital platforms [11]. However, prospective studies evaluating interventions delivered entirely through eHealth, incorporating continuous remote monitoring and structured clinician feedback, remain limited.

Therefore, this pilot study aimed to evaluate the safety, participant adherence, and preliminary clinical efficacy of delivering a structured TDR program entirely through an eHealth platform to achieve substantial weight loss and type 2 diabetes remission.

## Methods

### Study Design and Participants

This single-arm, nonblinded, single-center pilot study evaluated a low-calorie diet delivered via an eHealth platform and had a study duration of 6 months. The 2 prespecified coprimary outcomes were weight loss of at least 15 kg and diabetes remission, defined as HbA_1c_ below 6.5% without the use of blood glucose-lowering medication. These coprimary outcomes were selected in accordance with the DiRECT trial, in which weight loss of 15 kg was chosen because previous evidence suggested that this magnitude of weight loss is strongly associated with type 2 diabetes remission. For individuals with a baseline body weight of less than 80 kg, the weight-loss target was 15% of baseline body weight, rather than 15 kg. For participants in whom further weight loss would have resulted in a BMI below 23 kg/m^2^, weight loss was discontinued, and weight maintenance was recommended.

The findings of this study are intended to inform the design of a subsequent randomized controlled trial comparing eHealth-based and face-to-face care.

This pilot study was conducted in Umeå, Sweden, between April and December 2021 and is reported in accordance with the CONSORT (Consolidated Standards of Reporting Trials) 2010 checklist for reporting pilot and feasibility trials (Checklist 1) [12].

Study participants were recruited through advertisements in local newspapers. Additionally, primary care physicians, diabetes nurses, and dietitians were informed about the study at local diabetes meetings and were encouraged to inform their patients about the possibility of participating. Study participants were not involved in the design or conduct of our research.

Eligible participants had been diagnosed with type 2 diabetes within the previous 6 years, were aged 20 to 70 years, had a BMI over 27 kg/m^2^, and had an HbA_1c_ of 6.5% to 11.9% during the last 12 months without diabetes medication. Exclusion criteria were insulin treatment, weight loss of more than 5 kg in the last 6 months, treatment with weight-loss medications, a diagnosed eating disorder, estimated glomerular filtration rate below 30 mL/min/1.73 m^2^, substance abuse, known cancer, myocardial infarction within the previous 6 months, severe heart failure (New York Heart Association [NYHA] class III), pregnancy or planned pregnancy, and treatment with antipsychotic drugs. Participants were asked to self-report the presence of neuropathy or retinopathy. After study initiation, the upper age limit was revised from 65 to 70 years to enable enrollment of 10 participants. No interim analyses were planned or conducted.

Before study initiation, we decided to include 10 participants in this pilot study.

### Intervention

The study participants replaced all regular food with the TDR formula Modifast (820‐890 kcal/d) for 12 weeks. To counteract constipation, psyllium husk was used as a dietary supplement as needed.

After 12 weeks of TDR, participants entered a structured food reintroduction phase (weeks 12‐18). During this phase, 1 TDR meal at a time was reintroduced.

These were gradually replaced with regular food based on participant preference. The dietary plan included 4 meals per day, with portion guidance provided for breakfast, lunch, dinner, and snacks. The target daily energy intake was 1500 to 1600 kcal for women and 1900 to 2100 kcal for men. Participants received dietary counseling focused on selecting whole grain products, low-fat dairy, foods labeled with the green keyhole symbol, unprocessed meats, and a high intake of vegetables, fruits, and berries. Guidance was also provided on managing potential increases in appetite during this transition. In the subsequent weight maintenance phase (weeks 19‐26), participants followed a fully food-based diet with individualized energy prescriptions from a dietitian to support weight stability. Some participants opted to continue replacing 1 daily meal with a TDR product during this phase.

All participants received a scale (*UC-325BLE Continua Body Scale* from A&D Medical, Ann Arbor, MI, USA), a finger-stick blood glucose meter (*Accu-Chek Instant* from Roche, Basel, Switzerland), and a blood pressure monitor (*UA-651BLE Continua Arm Blood Pressure Monitor* from A&D Medical, Ann Arbor, MI, USA). Participants were asked to measure body weight, blood glucose, and blood pressure every morning. The scale, glucose meter, and blood pressure monitor transmitted their measurements via Bluetooth to the *Telia Health Monitoring* mobile phone app on the study participant’s mobile phone. From there, the data were sent to the project’s physician, nurse, and dietitian. The results of the measurements were checked by the research nurse every weekday using the application’s secure website for result management. When action was required due to abnormal measurement results, participants received feedback directly or the information was passed on to the doctor or dietitian for follow-up. The participants had a scheduled video visit with the study physician every 2 weeks for the first 3 months. Thereafter, study participants met the study dietitian every 2 weeks until month 4 and then once a month until the end of the study. During the video visits, home measurements of body weight, fasting blood glucose, and blood pressure were discussed.

At the beginning of the food reintroduction phase, participants received an activity tracker (*Vivosmart 4 Activity Tracker* from *Garmin*, Olathe, Kansas) that worked together with the *Telia Health Monitoring* app. During the 12 weeks of TDR, participants were advised not to increase their physical activity. At the beginning of the food reintroduction phase, participants were advised to reach and maintain their sustainable maximum of up to 15,000 steps per day.

HbA_1c_ was measured monthly using finger-prick blood samples collected by participants at home as part of the intervention. The samples were sent via regular mail to the Department of Clinical Chemistry at Norrland University Hospital for analysis. The HbA_1c_ results were subsequently reviewed and discussed with study personnel during scheduled video consultations.

All diabetes and blood pressure medications were discontinued on the day the TDR was started because blood glucose levels fall fast, and blood pressure is likely to fall with strict calorie restriction. Medication was reintroduced, as described below, based on blood pressure, blood glucose, and HbA_1c_ measurements.

### Treatment of High Blood Pressure

The study participants were instructed to avoid salt intake. Blood pressure medication was restarted if systolic blood pressure was over 140 mm Hg on repeated measurements (over 165 mm Hg during the first 2 wk of TDR). To simplify treatment decisions, only systolic blood pressure was used.

### Treatment for High Blood Glucose

Oral hypoglycemic agents were considered only when participants had intolerable hyperglycemic symptoms (polyuria and polydipsia) or a random blood glucose level above 20 mmol/L. If insufficient weight loss led to worsening glycemia, adherence to the low-calorie diet was addressed. If weight loss was adequate but glycemic control remained unsatisfactory, initiation of oral hypoglycemic agents was considered.

### Rescue Plan

If a weight gain of more than 2 kg occurred during the weight loss maintenance phase, diet replacement was recommended for 1 to 2 meals per day for 4 weeks, and a prescription for orlistat was offered. In cases of a weight gain of more than 4 kg or a total weight loss of less than 15 kg from the starting weight, or if diabetes returned, TDR for all meals was recommended for 4 weeks, and thereafter regular meals were reintroduced for 2 to 4 weeks. In addition, orlistat was offered.

### Outcome Measures

All outcome measurements were performed at baseline before initiation of the intervention and at 3 and 6 months at the Clinical Research Center, Umeå University Hospital. Weight was measured on the same scale in light clothing without shoes, and length on a digital height-measuring gauge. Waist circumference was measured midway between the iliac crest and the lower rib. Blood pressure was measured in the right arm with an automatic blood pressure monitor after 5 minutes of rest. Three blood pressure readings were obtained; the first was deleted, and the average pressure of the other 2 was registered.

Fasting venous blood samples were collected and analyzed at the Department of Clinical Chemistry, Umeå University Hospital, using routine clinical laboratory methods.

B-HbA_1c_ was analyzed using high-performance liquid chromatography with the TOSOH G11 system. Fasting plasma glucose was analyzed by spectrophotometry using the Cobas Pro c503 system and GLUC3 reagent (Roche Diagnostics Scandinavia AB). Glycemic status was classified according to WHO criteria as follows: diabetes, fasting plasma glucose greater than or equal to 126 mg/dL (7.0 mmol/L); impaired fasting glucose (IFG): fasting plasma glucose 110 to 125 mg/dL (6.1‐6.9 mmol/L). Plasma insulin and C-peptide were analyzed by immunometry using the Cobas Pro e801 with Insulin and C-peptide reagents (Roche Diagnostics). The lipid profile, including plasma cholesterol, triglycerides, and high-density lipoprotein cholesterol, was analyzed by spectrophotometry using the Cobas Pro c503 with Cholesterol Gen.2 (CHOL2), Triglycerides (TRIGL), and HDL-Cholesterol Gen.4 (HDLC4) reagents, respectively. Low-density lipoprotein cholesterol was calculated using the Friedewald formula. Liver enzymes, alanine transaminase (ALT) and aspartate aminotransferase (AST), were analyzed by spectrophotometry using the Cobas Pro c503 analyzer with Alanine Aminotransferase acc. to IFCC II (ALTP2) and Aspartate Aminotransferase acc. to IFCC II (ASTP2) reagents, respectively. C-reactive protein (CRP) was analyzed by immunoturbidimetry using the CRP4 reagent (Roche Diagnostics).

An oral glucose tolerance test (OGTT) was performed in the fasting state, during which participants ingested 75 g of glucose dissolved in 200 mL of Topstar solution. Glucose, insulin, and C-peptide levels were measured at 0, 30, 60, 90, and 120 minutes. Glucose tolerance was classified according to the WHO criteria as follows: diabetes, 2-hour plasma glucose greater than or equal to 200 mg/dL (11.1 mmol/L); impaired glucose tolerance (IGT): 2-hour plasma glucose 140-199 mg/dl (7.8‐11.0 mmol/L).

Respiratory quotient (RQ) and resting energy expenditure (REE) were measured using indirect calorimetry (Quark resting metabolic rate, COSMED Nordic ApS). Assessments were performed after an overnight fast, with participants in a supine position. Measurements were obtained 30 minutes before the OGTT, following 20 minutes of rest, and during the final 30 minutes of the OGTT. During each measurement, participants lay under a ventilated hood while the volume of oxygen consumption (VO_2_) and the volume of carbon dioxide production (VCO_2_) were recorded. The last 10 minutes of each 30-minute recording period were used to estimate RQ and REE, with the requirement that variability during this interval was less than 10% to ensure measurement stability. The RQ was calculated as the ratio of VCO_2_ to VO_2_, with expected values ranging between 0.7 and 1.0, indicating predominant fat oxidation at lower values (RQ≈0.7) and predominant carbohydrate oxidation at higher values (RQ≈1.0). Metabolic flexibility was assessed by comparing fasting RQ values to RQ values following glucose ingestion, reflecting the body’s ability to shift from fat to carbohydrate oxidation. REE was calculated from fasting measurements using the Weir equation: REE (kcal/d) = 1440 × (3.941 × VO_2_ [L/min] + 1.106 × VCO_2_ [L/min]). No changes to the measurements were made after the pilot study began.

### Statistical Analysis

Data are presented as mean (SD) or median (IQR). The trapezoidal rule was used to calculate the area under the curve (AUC). Data over time from baseline to 6 months were analyzed using the Wilcoxon signed-rank test. A 2-sided *P* value of <.05 was considered statistically significant. All analyses were performed using SPSS Statistics (version 28.0; IBM Corp).

### Ethical Considerations

Ethical approval was granted by the Swedish Ethical Review Authority (Dnr 2021‐00318). The study was prospectively registered at ClinicalTrials.gov (NCT04805996).

## Results

A total of 43 individuals were assessed for eligibility in this 6-month pilot study; 33 were excluded, of whom 26 did not meet the inclusion criteria and 7 declined to participate. Ten individuals were enrolled and completed the study. Baseline characteristics are summarized in Table 1.

The prespecified coprimary outcome, weight loss of at least 15 kg at 6 months, was achieved by 6 of 10 study participants. Mean body weight decreased by 16.9 (SD 5.4) kg among all 10 participants between baseline and 6 months (*P*=.005; Figure 1).

**Table 1.** Baseline characteristics.

Characteristic	Participants (N=10)
Women and men, (n)	1 and 9
White ethnicity, (n)	10
Age (y), mean (SD)	58.5 (7.4)
BMI (kg/m^2^), mean (SD)	35.3 (5.9)
Waist (cm), mean (SD)	122.1 (12.2)
Systolic blood pressure (mm Hg), mean (SD)	136.8 (11.4)
Diastolic blood pressure (mm Hg), mean (SD)	85.5 (9.1)
Diabetes duration (y), mean (SD)	2.8 (1.3)
HbA_1c_^a^ (%), mean (SD)	6.8 (0.8)
Fasting glucose (mmol/L), mean (SD)	8.4 (1.6)
Oral diabetes medication, (n)	
Metformin	10
Sulfonylurea	1
Antihypertensive medication, (n)	
Hypertension medication	7
ACE^b^ inhibitor	3
ARB^c^	2
Calcium-channel blocker	2
Beta-blocker	5
Thiazide	4
Statin, (n)	6
Albuminuria^d^, (n)	1
Retinopathy^e^, (n)	0
Neuropathy^e^, (n)	1
Type 2 diabetes heredity, (n)	5

a HbA_1c_: glycated hemoglobin.

b ACE: angiotensin-converting enzyme.

c ARB: angiotensin-receptor blocker.

d Defined as an albumin-to-creatinine ratio of ≥3 mg/mmol.

e Participants were only asked to self-report the presence of neuropathy or retinopathy.

**Figure 1.**
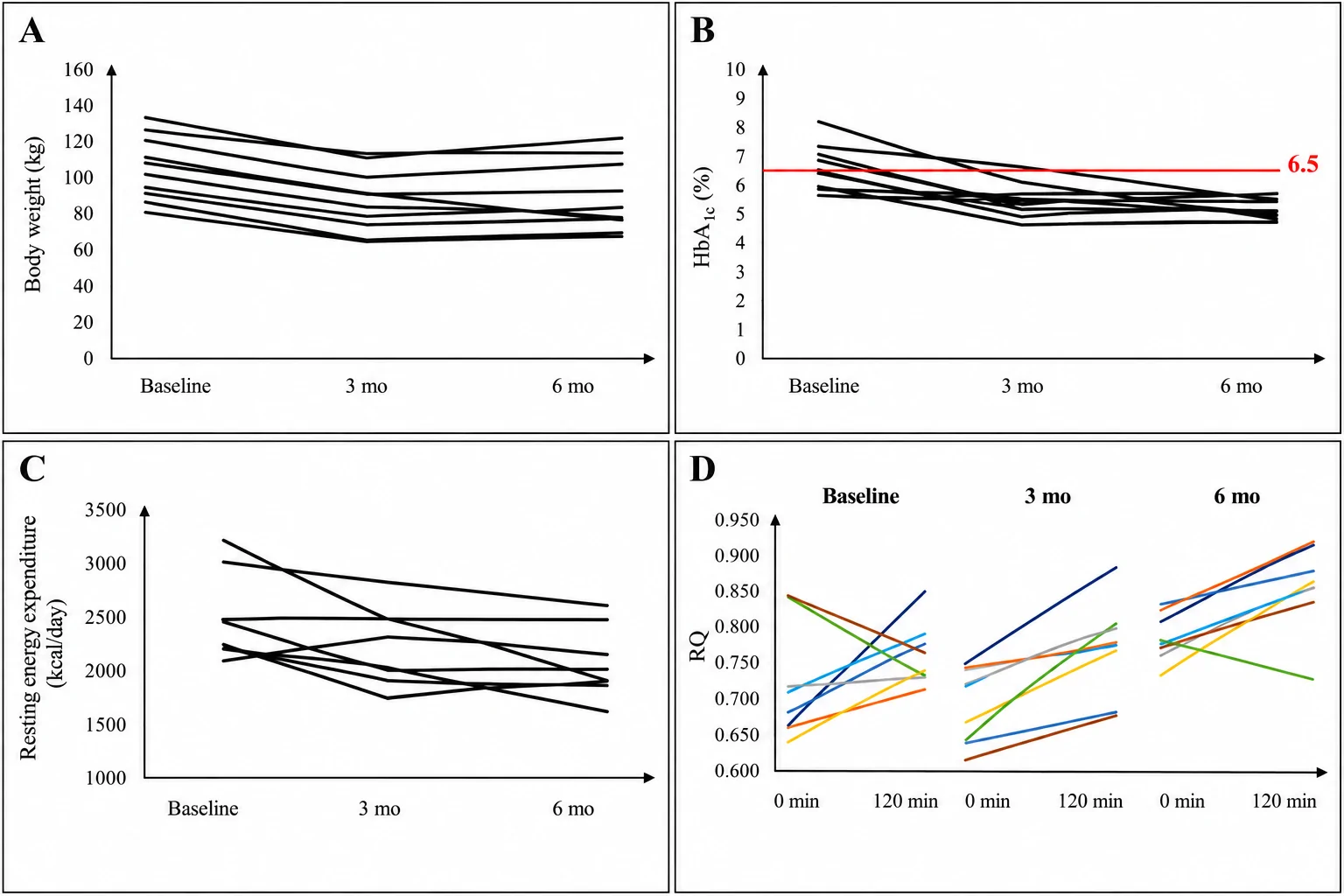
(A) Body weight in 10 individuals with type 2 diabetes using total diet replacement (TDR) for weight loss for 3 months, followed by food reintroduction and a weight loss maintenance phase; follow-up was exclusively by eHealth. (B) Hemoglobin (HbA_1c_) in individuals with type 2 diabetes using TDR for weight loss for 3 months, followed by food reintroduction and a weight loss maintenance phase; follow-up was conducted exclusively by eHealth. One of the prespecified coprimary outcomes was diabetes remission (HbA_1c_<6.5% without diabetes medication) at 6 months. All study participants were treated with metformin at recruitment, which was discontinued at study start. (C) Resting energy expenditure at study start (baseline), after the TDR phase (3 mo), and after food reintroduction in the weight maintenance phase (6 mo) in 8 individuals with type 2 diabetes being supported by eHealth. Data are mean (SD). (D) Respiratory quotient (RQ) in the fasting state (0 min) and after 75 g glucose (120 min) at study start (baseline), after the TDR phase (3 mo), and after food reintroduction in the weight-loss maintenance phase (6 mo) in 8 individuals with type 2 diabetes. Different colored lines represent individual participants and are used to distinguish their RQ responses.

The prespecified coprimary outcome, diabetes remission (HbA_1c_<6.5% without diabetes medication), was achieved by all 10 participants at 6 months (Figure 1). All participants were treated with at least metformin at recruitment (Table 1) and discontinued their diabetes medication at the start of the study. Despite the change in medication, HbA_1c_ decreased by a mean of 1.0% (SD 0.8%) from baseline to 6 months (*P*=.008). None of the study participants restarted any diabetes medication during the 6 months of the study.

Fasting plasma glucose, measured at the research facilities, decreased during the study (Table 2). At 6 months, 4 participants had normal fasting glucose, 5 had IFG, and 1 had a fasting glucose in the diabetic range. The median plasma glucose level at 120 minutes after the glucose bolus decreased from 12.4 mmol/L (IQR 10.3‐16.8) at baseline to 9.5 mmol/L (IQR 9.2‐12.1) after 6 months (*P*=.01 for the change over time; Figure 2). At 6 months, 3 participants had diabetic-range glucose levels at 120 minutes after the glucose bolus, while 7 participants exhibited IGT. The AUC for glucose after the OGTT decreased by a median of 26% (IQR 20%-32%) between baseline and 6 months (*P*=.009 for the change over time).

Fasting plasma insulin and C-peptide decreased between baseline and 6 months (Table 2). The AUC for insulin decreased by a median of 20% (IQR 3%-40%) between baseline and 6 months (*P*=.047 for the change over time; Figure 2). The AUC for C-peptide decreased by a median of 16% (IQR 10%-28%) between baseline and 6 months (*P*=.007 for the change over time; Figure 2).

**Table 2.** Metabolic, hormonal, and lipid parameters during a 6-month weight-loss intervention in individuals with type 2 diabetes.

Variable	n	Baseline, median (IQR)	3 months, median (IQR)	*P* value^a^	6 months, median (IQR)	*P* value^b^
Fasting glucose (mmol/L)	10	8.4 (7.0‐9.0)	5.9 (5.4‐6.6)	.005	6.1 (6.0‐6.5)	.007
Fasting insulin (mIU/L)	10	21.0 (13.7‐39.3)	14.0 (8.4‐18.0)	.005	11.0 (7.7‐16.3)	.007
Fasting c-peptide (nmol/L)	10	1.4 (1.1‐1.9)	1.1 (1.0‐1.3)	.008	0.9 (0.8‐1.2)	.007
Glucose AUC^c^ (mmol/L × min)	10	1501 (1316‐1825)	1078 (968‐1266)	.07	1191 (1084‐1303)	.009
Insulin AUC (mIU/L × min)	10	9353 (4440‐16103)	6375 (4554‐8704)	.04	5089 (3173‐11269)	.047
C-peptide AUC (nmol/L × min)	10	322 (241‐462)	294 (268‐349)	.33	266 (217‐336)	.007
Insulinogenic index	10	13 (4‐22)	12 (6‐27)	.58	11 (6‐28)	.72
Matsuda index	10	23 (16‐44)	43 (35‐75)	.04	68 (35‐91)	.005
Resting energy expenditure (kcal/d)	8	2216 (2083‐2708)	2007 (1769‐2293)	.03	1791 (1701‐2151)	.01
RQ^d^ (VCO_2_^e^ to VO_2_^f^) 0 min	8	0.67 (0.64‐0.80)	0.67 (0.63‐0.72)	.89	0.78 (0.77‐0.82)	.05
RQ (VCO_2_ to VO_2_) 120 min	8	0.74 (0.72‐0.77)	0.75 (0.68‐0.78)	>.99	0.86 (0.84‐0.90)	.01
CRP^g^ (mg/L)	10	1.6 (0‐2.9)	2.3 (0‐3.9)	.17	1.2 (0‐2.9)	.17
Total cholesterol (mmol/L)	10	3.0 (2.9‐5.1)	4.0 (2.7‐5.0)	.64	3.3 (3.0‐4.5)	>.99
Triglycerides (mmol/L)	10	2.1 (1.3‐3.4)	1.5 (1.0‐1.8)	.005	1.6 (1.1‐1.9)	.03
HDL^h^ cholesterol (mmol/L)	10	1.0 (0.9‐1.2)	1.1 (0.9‐1.3)	.31	1.2 (1.0‐1.3)	.11
LDL^i^ cholesterol (mmol/L)	10	1.3 (1.2‐2.8)	2.3 (1.2‐3.0)	.07	1.8 (1.3‐2.6)	.26
ALAT^j^ (ukat/L)	10	0.53 (0.35‐0.69)	0.39 (0.28‐0.54)	.59	0.34 (0.27‐0.45)	.09
ASAT^k^ (ukat/L)	10	0.46 (0.32‐0.54)	0.36 (0.35‐0.53)	>.99	0.34 (0.28‐0.42)	.24

a *P* value for baseline vs 3 months.

b *P* value for baseline vs 6 months.

c AUC: area under the curve.

d RQ: respiratory quotient.

e VCO_2_: volume of carbon dioxide production.

f VO_2_: volume of oxygen consumption.

g CRP: C-reactive protein.

h HDL: high-density lipoprotein.

i LDL: low-density lipoprotein.

j ALT: alanine aminotransaminase.

k AST: aspartate aminotransferase.

**Figure 2.**
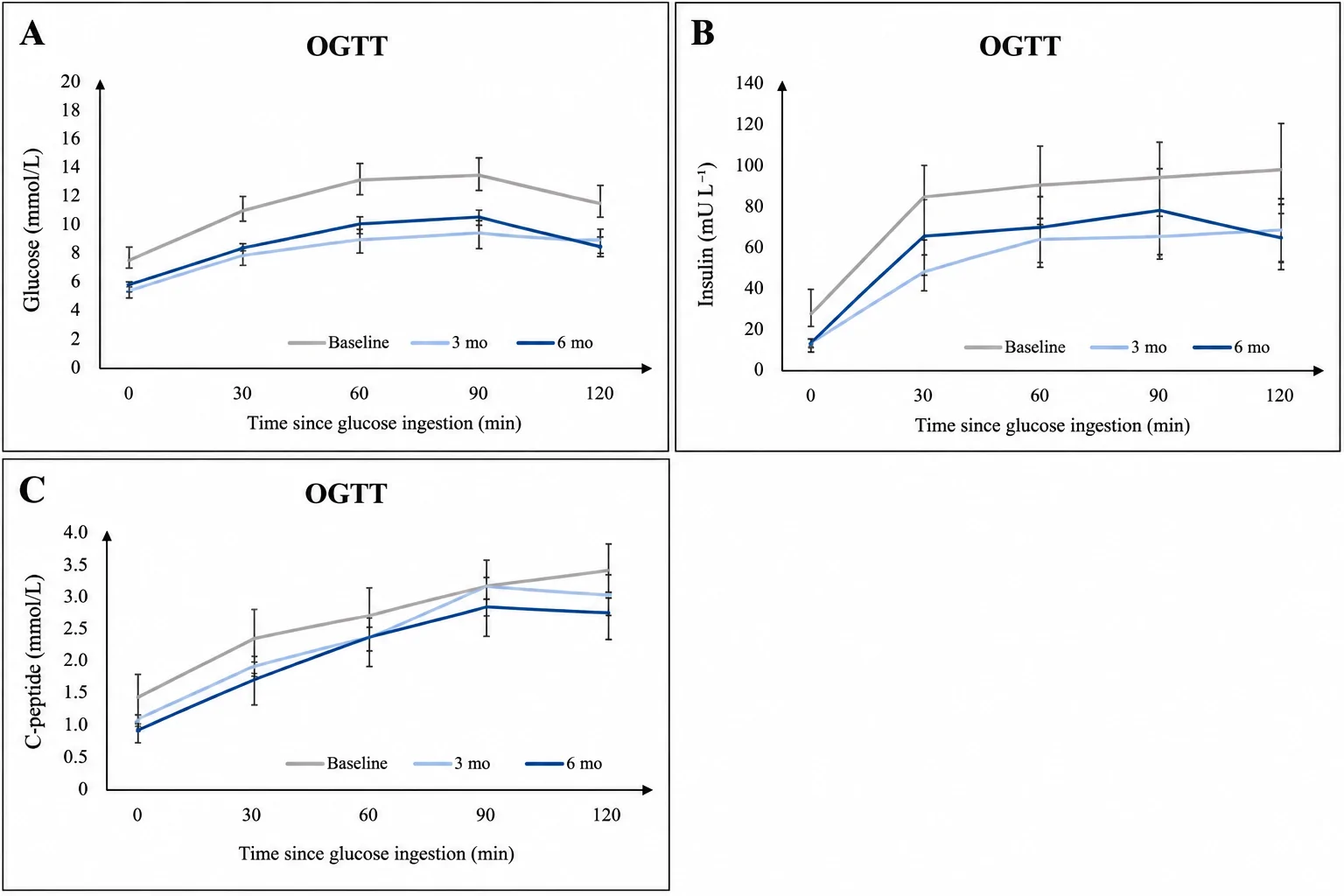
(A) Plasma glucose after 75 g glucose at study start (baseline), after the total diet replacement (TDR) phase (3 mo), and after food reintroduction in the weight maintenance phase (6 mo) in individuals with type 2 diabetes supported by eHealth. Data are mean (SD). (B) Plasma insulin after 75 g glucose at study start (baseline), after the TDR phase (3 mo), and after food reintroduction in the weight maintenance phase (6 mo) in individuals with type 2 diabetes supported by eHealth. Data are mean (SD). (C) Plasma C-peptide after 75 g glucose at study start (baseline), after the TDR phase (3 mo), and after food reintroduction in the weight maintenance phase (6 mo) in individuals with type 2 diabetes supported by eHealth. Data are mean (SD). OGTT: oral glucose tolerance test.

Daily home measurements of body weight, expressed as a percentage change from baseline, are depicted in Figure 3. During the initial 3 months, 9 participants experienced a weight reduction of 10% to 15%. In the subsequent 3 months, 2 participants continued to lose weight, whereas the remaining participants either maintained their reduced weight or exhibited a modest weight regain of up to 5%. Daily home measurements of fasting glucose showed a marked reduction in fasting glucose levels that occurred during the first week of TDR (Figure 3).

**Figure 3.**
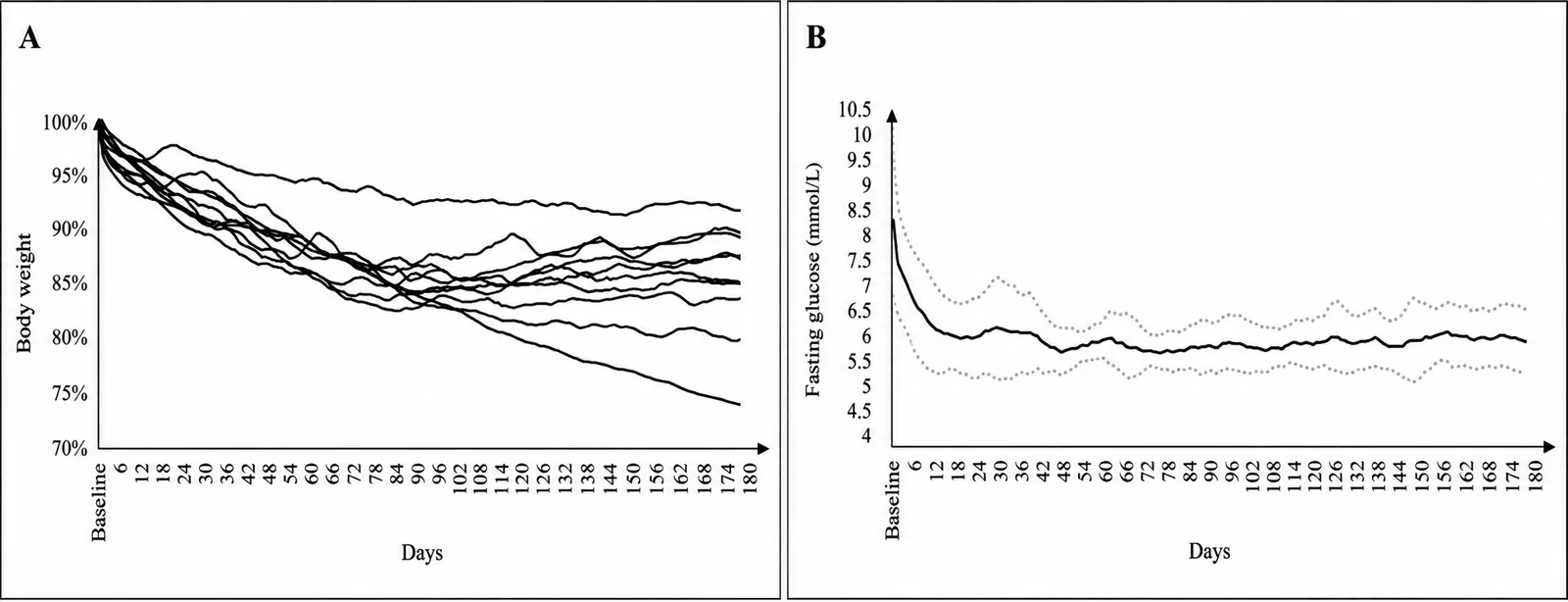
(A) Body weight (depicted as % change from baseline weight) based on daily home measurements of each study participant over a study duration of 180 days. (B) Fasting capillary glucose based on daily home measurements of 10 study participants with type 2 diabetes over a study duration of 180 days. The solid black line represents the mean fasting glucose, and the gray dotted lines represent standard deviation (SD).

Insulin sensitivity, evaluated using the Matsuda index, increased by a median of 100% (IQR 84%-166%; *P*=.005 for the change from baseline to 6 mo, Table 2). However, insulin secretion, assessed by the insulinogenic index, did not change significantly over the 6 months of the study.

REE and RQ were evaluated in 8 participants; however, data from 2 participants were unavailable due to technical issues with the indirect calorimetry equipment. Changes in RQ and REE over the study period are summarized in Table 2. The median decrease in REE from baseline to 6 months was 387 (IQR 381-536) kcal/d; (*P*=.01 for the change over time; Figure 1). Fasting RQ increased by 15% (IQR 5%-27%), and postglucose RQ increased by 12% (IQR 9%-18%) from baseline to 6 months (Figure 1).

At baseline, 6 participants demonstrated an increase in RQ following glucose ingestion, whereas 2 showed a paradoxical decrease in RQ at 120 minutes compared to fasting. At 6 months, 1 of these 2 participants exhibited a paradoxical reduction in RQ after glucose ingestion, while the other displayed a normal postglucose RQ increase.

During the initial weeks of the TDR phase, several transient adverse effects were reported. Of the 10 participants, the most common were headache (n=6, 60%), orthostatic hypotension (n=6, 60%), dizziness (n=5, 50%), and constipation (n=4, 40%). A few participants experienced episodes of hypoglycemia (n=2, 20%) or hyperglycemia (n=1, 10%), and 1 participant reported diarrhea (n=1, 10%). These adverse effects occurred primarily during the early adaptation period and resolved spontaneously or improved with increased fluid intake and supportive guidance without leading to study discontinuation. One participant was unable to continue the TDR beyond the first month because of other health-related issues and was therefore transitioned to a lower-calorie food-based diet.

## Discussion

### Diabetes Remission and Weight Loss Outcomes

This pilot study evaluated whether a TDR intervention supported by eHealth could induce clinically meaningful weight loss and achieve diabetes remission in individuals with type 2 diabetes. Of 10 participants, 60% (n=6) achieved the coprimary outcome of at least 15 kg of weight loss, while the remaining 40% (n=4) lost more than 10 kg. At 6 months, all participants achieved the coprimary outcome of diabetes remission without glucose-lowering medication.

In this study, daily home monitoring of body weight and fasting glucose provided real-time data, enabling the study team to identify insufficient weight loss or weight regain and provide timely support. Daily fasting glucose monitoring also allowed early detection of deteriorating glycemic control, making discontinuation of all glucose-lowering medication a safe component of the intervention.

The exclusive use of eHealth support was associated with high engagement and adherence, consistent with previous studies showing that eHealth interventions improve weight loss and glycemic control. A systematic review found eHealth interventions more effective than control interventions or no care, and comparable to face-to-face interventions for promoting weight loss and weight maintenance [9].

Regarding diabetes remission, defined as HbA_1c_ below 6.5% without glucose-lowering medication, our findings are consistent with previous studies showing that substantial weight loss is the primary driver of diabetes remission, particularly among individuals with a shorter duration of type 2 diabetes. The Counterpoint study first demonstrated that intensive calorie restriction could restore normoglycemia in individuals with recently diagnosed type 2 diabetes, while the subsequent Counterbalance study highlighted the importance of preserved β-cell function and shorter diabetes duration for achieving remission [13,14]. These findings were later confirmed in larger intervention trials. The DIADEM-I trial, which enrolled adults with type 2 diabetes of 3 years duration or less, reported a remission rate of 61% after 12 months. In comparison, the DiRECT trial included participants with type 2 diabetes of up to 6 years duration and reported remission in 57% (16/28) of those achieving weight loss of 10 to 15 kg and in 86% (31/36) of those losing more than 15 kg at 1 year [6,15]. In our study, all participants achieved diabetes remission after 6 months. Although the small sample size precludes direct comparison, the shorter intervention period, relatively short diabetes duration, and substantial weight loss may explain the higher remission rate.

According to HbA_1c_, all participants in our study achieved diabetes remission by the end of the study. However, fasting plasma glucose indicated normoglycemia in only 4 participants, IFG in 5, and diabetes in 1. Similarly, the OGTT showed diabetes in 3 participants and IGT in 7. This discrepancy between HbA_1c_ and glucose-based measures likely reflects the different aspects of glucose metabolism captured by each marker. HbA_1c_ reflects average glycemia over the preceding 8 to 12 weeks, fasting glucose primarily reflects hepatic glucose regulation, and 2-hour postload glucose levels provide insight into postprandial glucose metabolism and β-cell function. During early metabolic recovery, as seen in dietary intervention studies, hepatic insulin sensitivity and fasting glucose may normalize before β-cell function is fully restored, leading to persistently elevated 2-hour glucose levels despite normalized HbA_1c_ and fasting glucose. Previous studies have also shown that HbA_1c_ alone may underestimate glucose dysregulation, particularly in individuals with IGT [16,17]. Therefore, remission in this study should be interpreted according to consensus HbA_1c_ criteria rather than as complete normalization of glucose metabolism.

In our study, the greatest reduction in fasting glucose occurred during the first week, as demonstrated by daily home monitoring. Because this preceded substantial weight loss, it is likely to have been driven primarily by caloric restriction. Similar findings were reported by Lim et al [13], who showed that fasting glucose normalized within 7 days of severe caloric restriction (600 kcal/d), accompanied by normalization of hepatic glucose production and a marked reduction in hepatic insulin resistance.

Insulin sensitivity also improved markedly over the 6-month intervention. This contrasts with Lim et al [13], who reported no improvement in insulin sensitivity after 8 weeks of severe caloric restriction, with a mean weight loss of 15 (SD 4) kg among 11 individuals who achieved diabetes remission. However, our group has previously shown that 12 weeks of diet-induced weight loss improves peripheral insulin sensitivity in individuals with type 2 diabetes [18].

Although insulin sensitivity improved in our study, insulin secretion did not improve. This contrasts with Lim et al [13], who reported that diabetes remission was primarily driven by a rapid improvement in insulin secretion, evident within the first week and nearly normalized by week 8. A possible explanation is the different methods used to assess β-cell function. Lim et al [13] used a stepped insulin secretion test with arginine, a more direct and sensitive measure of β-cell function, whereas we used the insulinogenic index derived from OGTT glucose and insulin measurements, which may be less sensitive to subtle or early changes in β-cell responsiveness. Therefore, it remains unclear whether diabetes remission requires improvements in both insulin secretion and insulin sensitivity or whether improved insulin secretion alone is sufficient.

REE decreased progressively over the 6-month intervention, with a median reduction of 387 (IQR 381-536) kcal/day, accompanied by a mean weight loss of 16.9 (SD 5.4) kg. This exceeds the reductions reported in the POUNDS LOST study, where REE decreased by approximately 100 kcal/day in men and 55 kcal/day in women after mean weight losses of 7.6 (SE 0.34) kg and 5.89 (SE 0.27) kg, respectively, at 6 months [19].

The greater reduction in REE observed in our study may partly reflect the fact that postintervention assessments were performed immediately following the weight-loss phase, when some participants were likely still in negative energy balance. Consistent with this interpretation, Martins et al [20] showed that the apparent metabolic adaptation observed immediately after weight loss was substantially attenuated after weight stabilization, suggesting that part of the reduction in REE reflects ongoing negative energy balance rather than a persistent suppression of resting metabolism. Therefore, the reduction in REE observed in our study should be interpreted with caution, as it may partly represent a transient physiological response to active weight loss rather than a long-term change.

The RQ is the ratio of carbon dioxide produced to oxygen consumed during metabolism and provides insight into the balance between carbohydrate and fat oxidation [21,22]. An RQ of 1.0 indicates exclusive carbohydrate oxidation, whereas an RQ of 0.7 indicates exclusive fat oxidation, with intermediate values reflecting mixed substrate utilization. Metabolic flexibility refers to the ability to adapt fuel oxidation to fuel availability by switching between carbohydrate and fat oxidation [22,23]. Impaired metabolic flexibility is common in obesity and type 2 diabetes [24,25]. At baseline, the low fasting RQ (0.67) indicated predominant fat oxidation, while the modest increase after glucose ingestion (0.74) suggested impaired metabolic flexibility. After 6 months, fasting RQ increased to 0.78, indicating greater carbohydrate oxidation in the fasting state, contrary to our hypothesis. However, postglucose RQ increased more markedly to 0.86, consistent with improved metabolic flexibility. These findings are consistent with previous studies showing that improvements in insulin sensitivity following lifestyle intervention are accompanied by greater increases in RQ during glucose or meal stimulation rather than consistent changes in fasting RQ [26]. Together, our findings suggest that weight loss improved the capacity to oxidize carbohydrates when they were available. We did not observe increased fasting fat oxidation, possibly because fat oxidation was already high at baseline. In addition, some participants may still have been in negative energy balance at the postintervention assessment, which could have influenced the RQ measurements [27].

A key strength of this pilot study was the high level of participant engagement and adherence, supported by daily self-monitoring and continuous eHealth feedback from the study team. This real-time interaction likely contributed to sustained motivation and compliance. The diet intervention, including TDR, was evidence-based and designed to minimize barriers to adherence by reducing the need for food preparation, while clearly defined treatment goals promoted consistency.

Several limitations should be acknowledged. This pilot study included only 10 participants, all with relatively short-duration type 2 diabetes (mean 2.8, SD 1.3 y), which is known to increase the likelihood of achieving remission. The wide age range may have increased variability. Although *P* values are reported, the small sample size may have led to an overestimation of statistical significance. The intervention and follow-up were relatively short. Recruitment through local newspaper advertisements may have introduced selection bias by attracting highly motivated individuals, limiting generalizability to routine clinical populations. In addition, only 1 participant was a woman and none were from non-White ethnic backgrounds, limiting the generalizability of the findings. Women may face additional barriers to participating in intensive lifestyle interventions, including caregiving responsibilities, family commitments, time constraints, and logistical challenges [28,29]. Future randomized studies should therefore use targeted recruitment strategies, including recruitment through primary care and community outreach, flexible scheduling, and prespecified recruitment targets, to improve the representation of women and ethnically diverse populations and enhance external validity [28,30,31].

Glucagon-like peptide-1 receptor agonists and tirzepatide were not used, as pharmacologically induced weight loss would have precluded evaluation of medication-free diabetes remission. As incretin-based therapies become more widely used, digitally delivered dietary interventions may primarily appeal to individuals seeking medication-free remission or those with limited access to, or contraindications to, these therapies.

Given the increasing prevalence of obesity and type 2 diabetes, public health efforts should prioritize interventions that are both accessible and scalable. If eHealth-based interventions prove effective in larger and more diverse populations, they could provide a sustainable and cost-effective complement or alternative to in-person care while improving access for people living in rural or underserved communities.

### Conclusions

This pilot study demonstrates that type 2 diabetes of short duration may enter remission through substantial weight loss, as reflected by HbA_1c_ levels falling below 6.5% without the use of diabetes medication. However, long-term remission appears to depend on sustained weight loss, as relapse is likely with weight regain. While all participants met the HbA_1c_ criterion for remission, postload glucose levels from the OGTT remained in the impaired or diabetic range in several individuals, highlighting a discrepancy between HbA_1c_ and dynamic glucose regulation. This suggests that HbA_1c_ alone may underestimate residual glycemic impairment and that full metabolic recovery may not yet be complete. These findings underscore the potential of structured total replacement diet interventions, supported by eHealth tools, to achieve clinically meaningful remission in type 2 diabetes, even outside conventional health care settings. However, further research is needed to confirm these findings in larger and more diverse populations over a longer period.

## Supplementary material

10.2196/93074 Checklist 1 CONSORT 2010 checklist.

## Abbreviations:

ADA: American Diabetes Association
AUC: area under the curve
CONSORT: Consolidated Standards of Reporting Trials
CRP: C-reactive protein
DIADEM-I: Diabetes Intervention Accentuating Diet and Enhancing Metabolism-I
DiRECT: Diabetes Remission Clinical Trial
EASD: European Association for the Study of Diabetes
HbA_1c_: glycated hemoglobin
IFG: impaired fasting glucose
IGT: impaired glucose tolerance
NHS: National Health Service
NYHA: New York Heart Association
OGTT: oral glucose tolerance test
REE: resting energy expenditure
RQ: respiratory quotient
TDR: total diet replacement
VCO_2_: volume of carbon dioxide production
VO_2_: volume of oxygen consumption
WHO: World Health Organization
